# The Impact of a Public Awareness Campaign on Perceptions of Lung Cancer Risk Factors and Screening Guidelines

**DOI:** 10.3390/healthcare13131555

**Published:** 2025-06-30

**Authors:** Rayan A. Qutob, Lama Abdullah Alkhwildi, Amal Abdullah Alghtani, Tamadher Misfer Alsalouli, Arwa Saif Alarifi, Mohammed Salem M. Alshehri, Hessah Abdulrahman Almojel, Abdullah Alaryni, Eysa Alsolamy, Yousef Alammari, Abdulrahman Alanazi, Abdullah Alghamdi, Mohammad A. Alhajery, Khalid I. AlHussaini, Mosaad Almegren

**Affiliations:** 1 Department of Internal Medicine, College of Medicine, Imam Mohammad Ibn Saud Islamic University (IMSIU), Riyadh 11564, Saudi Arabia; dr.rayanq@hotmail.com (R.A.Q.); al3raini@hotmail.com (A.A.); eysa783@hotmail.com (E.A.); yalammari@gmail.com (Y.A.); amn654@gmail.com (A.A.); dr.alhomrani@gmail.com (A.A.); maalhajery@imamu.edu.sa (M.A.A.); kialhussaini@imamu.edu.sa (K.I.A.); mosaad966@gmail.com (M.A.); 2 College of Medicine, Imam Mohammad Ibn Saud Islamic University (IMSIU), Riyadh 11564, Saudi Arabia; lamalkhwildi@gmail.com (L.A.A.); amalaalghtani@gmail.com (A.A.A.); arwa-123@live.com (A.S.A.); hessah1422@hotmail.com (H.A.A.); 3 College of Medicine, Tabuk University, Tabuk 47512, Saudi Arabia; msalshehri10@gmail.com

**Keywords:** lung cancer, public awareness, health campaign, screening guidelines, risk factors, smoking cessation, low-dose CT (LDCT), cancer prevention, health education

## Abstract

**Introduction:** Lung cancer (LC) is the leading cause of cancer mortality and is responsible for 1.8 million deaths annually. The early identification of risk factors, particularly smoking, is essential in improving outcomes. Public health campaigns play a crucial role in raising awareness, but misinformation and resource limitations hinder their effectiveness. This study evaluates the impact of a public awareness campaign on Saudi citizens’ understanding of lung cancer (LC) risks and screening. **Methods:** An interventional study was conducted in Riyadh, Saudi Arabia, using pre- and post-campaign self-administered surveys. A total of 1,426 participants aged 18 or older were surveyed either before or after the campaign. A matching approach was used to control for confounding variables. Each participant may receive a maximum total score of 14 for their knowledge of lung cancer and a maximum total score of 10 for their awareness of lung cancer screening. **Results:** A total of 713 participants were surveyed pre-campaign, and 859 post-campaign). After matching, 308 participants remained for the analysis, with no significant demographic differences between those who were surveyed before and after the campaign. Post-campaign, awareness was significantly improved, which is reflected in an increase in accurate responses to key statements. The median knowledge scores increased from 11.0 to 23.0, indicating a substantial increase in understanding. **Conclusions:** The campaign effectively enhanced the awareness of LC risk factors and screening. However, new misconceptions regarding universal screening emerged, emphasizing the need for clear messaging. Future initiatives should address socioeconomic and gender disparities, promote collaborative decision-making, and implement long-term educational strategies. These findings align with previous research and highlight areas for improvement in public health outreach.

## 1. Introduction

Lung cancer (LC), a significant global health concern, ranks among the most common malignancies worldwide [1,2]. According to the World Health Organization, lung cancer (LC) is the leading cause of cancer mortality, and is responsible for approximately 1.8 million deaths annually. The early detection and an understanding of risk factors are crucial in reducing mortality rates, as they can significantly improve treatment outcomes [3]. Public awareness campaigns are vital in disseminating information about lung cancer (LC), its risk factors, and recommended screening guidelines, fostering a more informed public that can take proactive steps in their health management. Understanding the nuances of lung cancer risk factors and the importance of screening guidelines is essential. Research indicates that smoking is the primary risk factor for lung cancer, accounting for nearly 85% of all cases [4]. However, other factors, such as exposure to secondhand smoke, radon gas, and occupational hazards, also contribute significantly to the risks [5]. Studies suggest that many lung cancer (LC) cases could be prevented if public awareness campaigns have effectively addressed these risk factors. Additionally, low-dose computed tomography (LDCT) screening has been shown to reduce mortality in high-risk populations [6]. However, the awareness of both lung cancer (LC) risk factors and screening guidelines remains insufficient. This problem is further exacerbated by widespread misinformation, a lack of accessible resources, and low adherence to recommended screening practices [7]. While global efforts to educate the public on cancer prevention have achieved some success, many campaigns struggle to reach the most at-risk populations or fail to create lasting behavior changes. This emphasizes the need to assess the effectiveness of current public health campaigns and refine their approaches [8]. Although existing research has evaluated the impact of public awareness initiatives on other cancers, such as breast and colorectal cancers, there is a noticeable lack of studies focused on lung cancer (LC) [9]. This paucity is particularly concerning because lung cancer (LC) is often preventable through risk reduction and early detection. Evidence supports the effectiveness of educational interventions aimed at reducing the lung cancer risk. For instance, smoking cessation programs have demonstrated success in lowering the lung cancer incidence among high-risk individuals [10]. Similarly, public health campaigns that promote LDCT screening have improved early detection rates [6]. Nevertheless, few studies have examined whether these initiatives effectively reshape public perceptions. This study aims to evaluate the impact of a public awareness campaign on perceptions of lung cancer (LC) risk factors and screening guidelines. By exploring changes in knowledge and attitudes, this research seeks to bridge existing gaps, provide evidence-based insights into the effectiveness of awareness initiatives, and contribute to the development of strategies tailored to the needs of the Saudi population.

## 2. Methods

### 2.1. Study Design and Setting

This research employed an interventional study to investigate the knowledge and misconceptions surrounding lung cancer among residents in Riyadh, Saudi Arabia. We conducted a comprehensive literature review prior to this study; afterward, we submitted the initial proposal.Ethical approval was obtained in December 2024. This study’s data were collected over a two-day period in January 2025, using a self-administered survey distributed on-site, immediately after the campaign.

### 2.2. Ethical Approval

Institutional Review Board Statement: Ethical approval for this research was obtained from the Institutional Review Board (IRB) of Al-Imam Muhammad Ibn Saud Islamic University (project number 753/2024; approval date: 28 December 2024). All methods were implemented in accordance with the principles of the Declaration of Helsinki.

### 2.3. Participants and Sampling

Our study focused on a population of male and female residents in Riyadh, aged 18 to 50, encompassing both Saudi nationals and non-Saudis who consented to participate in this study. Individuals outside the specified age range or residing outside Riyadh were excluded. To ensure adequate statistical power and representativeness for our target population, we performed an a priori sample size calculation using the G*Power software (version 3.1.9.7). In this z-test for proportions (one-sample case), we assumed a conservative population proportion of 0.50, which maximizes the required sample size for a given margin of error. With a desired 95% confidence level (α = 0.05) and a 2.5% margin of error (0.025), the analysis indicated that a minimum sample size of 700 participants was necessary to reliably estimate awareness and knowledge levels.

### 2.4. Data Collection Procedures

Participants were recruited through convenience sampling during an awareness campaign event held in a prominent shopping mall in Riyadh. They provided explicit electronic consent before proceeding with the questionnaire. The questionnaire was administered on-site immediately following the conclusion of the awareness campaign sessions, typically within 5 min of participation. The survey was structured into two primary parts. The first part included questions related to sociodemographic characteristics such as age, gender, education, and smoking status. The second part comprised a series of statements related to lung cancer, to which participants responded by selecting “Agree”, “Disagree”, or “I do not know”. These statements were designed to assess misconceptions and knowledge about lung cancer risk factors and screening, and the questionnaire was completed by the participants either before or after joining the awareness campaign. Specifically, 713 participants completed the questionnaire prior to the campaign, while 859 completed it afterward. Due to the challenges in tracing participants across these two time points, matching was necessary to control for potential confounders and ensure comparability between the two groups.

### 2.5. Matching Procedure

To address the imbalance in participants who completed the questionnaire before and after the campaign, a matching procedure was implemented using the MatchIt package (version 4.5.3) in R The propensity score was estimated using a generalized linear model (GLM) with a logit link, with the matching variables including gender, age, education, and smoking status. Nearest-neighbor matching was applied with a caliper of 0.1, and observations lacking suitable matches in either group were discarded. This approach aimed to minimize the influence of confounding factors and isolate the effect of the awareness campaign on participants’ knowledge and misconceptions regarding lung cancer.

### 2.6. Study Questionnaire and Validation

The questionnaire was initially developed in English to align with standard survey research practices and subsequently translated into Arabic to accommodate the linguistic preferences of the target population. To ensure the accuracy and reliability of the translation, a rigorous back-translation process was conducted, wherein the Arabic version was translated back into English to verify that it faithfully represented the original content.

Following translation, content validity was established by 3 public health professionals. This panel assessed the relevance, clarity, and comprehensiveness of each item in relation to the study’s objectives. Prior to the main data collection procedure, a pilot study was conducted with a small, representative sample of 20 participants from the target population. Feedback from this pilot phase was instrumental in refining the questionnaire; enhancing the question clarity, cultural sensitivity, and linguistic precision; and optimizing the estimated completion time.

### 2.7. Knowledge Scoring

A knowledge scoring system was implemented to quantify participants’ understanding and identify misconceptions regarding lung cancer. One point was awarded for each correct response, with a maximum total score of 24. For knowledge regarding lung cancer, a maximum total score of 14 was possible. Moreover, for awareness regarding lung cancer screening, a maximum total score of 10 was possible.

### 2.8. Statistical Analysis

The data collected from the survey were exported to Microsoft Excel for initial organization and then analyzed using R v4.3. Quantitative data were summarized as means and standard deviations for continuous normal variables, and for non-normal variables, medians with interquartile ranges were used, while categorical data were expressed as percentages and counts. The chi-squared test was employed to examine the relationships between categorical variables. The Mann–Whitney U test was used to compare non-normal continuous data, and an unpaired *t*-test was applied for continuous normal data. The matching procedure using the MatchIt package (Version 4.5.3) in R ensured that the comparison between the pre-campaign and post-campaign groups was unbiased by controlling for confounding variables. Cronbach’s alpha was used to assess the reliability of the items for the individual domains and the total knowledge score, with scores greater than 0.7 considered satisfactory. Linear regression was employed to assess factors associated with knowledge before and after the campaign, with hypothesis testing performed at a 5% level of significance.

## 3. Results

A total of 1426 participants were recruited in this study, with 713 in the pre-campaign group and 859 in the post-campaign group. After applying propensity score matching using nearest-neighbor matching with a caliper of 0.1, 308 participants (154 from each group) were successfully matched based on gender, age, education, smoking status, and region (Table 1). The remaining 1118 participants (559 from each group) were unmatched and excluded from the final analysis due to differences in propensity scores and the absence of suitable counterparts within the caliper range. The unmatched sample exhibited significant differences across demographic variables, as shown in Appendix A.

The demographic analysis of the 154 matched participants in both the pre-campaign and post-campaign groups showed no statistically significant differences across key variables. The gender distributions were similar, with 1.3% of participants being female in the post-campaign group and 2.6% in the pre-campaign group (*p* = 0.684). The age distribution was comparable, with 35.1% of participants aged 20–29, 63.6% aged 30–39, and 1.3% aged 40–49 in the post-campaign group, while, in the pre-campaign group, 36.4% were aged 20–29 and 63.6% were aged 30–39 (*p* = 0.547).

Educational levels were similarly distributed, with 0.65% of the post-campaign group having a secondary education and 99.4% holding a bachelor’s degree, compared to 1.95% and 98.1%, respectively, in the pre-campaign group (*p* = 0.623). Smoking status was similar between the two groups, with 35.1% reporting no smoking history and 64.9% being smokers (*p* = 1.000). The monthly income distributions were similar, with 63.0% earning less than SAR 5000 in the post-campaign group, compared to 64.3% in the pre-campaign group, with similar distributions across other income categories were also observed (*p* = 0.819). The demographic characteristics for the unbalanced data are shown in Appendix A.

**Table 1:** Descriptive statistics for the study sample.

	Before Joining the Campaign	After Joining the Campaign	*p*
	N = 154	N = 154	
Gender:			0.684
Female n (%)	4 (2.60%)	2 (1.30%)	
Male n (%)	150 (97.4%)	152 (98.7%)	
Age:			0.547
20–29 n (%)	56 (36.4%)	54 (35.1%)	
30–39 n (%)	98 (63.6%)	98 (63.6%)	
40–49 n (%)	0 (0.00%)	2 (1.30%)	
Education:			0.623
Secondary n (%)	3 (1.95%)	1 (0.65%)	
Bachelor’s n (%)	151 (98.1%)	153 (99.4%)	
Smoking:			1.000
No n (%)	54 (35.1%)	54 (35.1%)	
Yes n (%)	100 (64.9%)	100 (64.9%)	
Monthly income:			0.819
< SAR 5000, n (%)	99 (64.3%)	97 (63.0%)	
SAR 5000–10,000, n (%)	54 (35.1%)	54 (35.1%)	
SAR 11,000–20,000, n (%)	1 (0.65%)	1 (0.65%)	
SAR 21,000–30,000, n (%)	0 (0.00%)	2 (1.30%)	

Data were summarized using counts and percentages. The chi-square test of independence was used for statistical testing. <SAR 5000: <USD 1350; 5000–10,000: USD 1350–2700; SAR 11,000–20,000: USD 2970–5400; SAR 21,000–30,000: USD 5670–8100.

There were significant differences in correct response rates between the pre-campaign and post-campaign groups for multiple survey items (Table 2). The awareness of lung cancer as one of the most common cancers increased from 0.65% to 100% after the campaign (*p* < 0.001). The recognition of shisha smoking (36.4% to 99.4%, *p* < 0.001), e-cigarette smoking (0.65% to 99.4%, *p* < 0.001), exposure to asbestos (34.4% to 100%, *p* < 0.001), radon gas (36.4% to 100%, *p* < 0.001), and air pollution (27.9% to 100%, *p* < 0.001) as risk factors also significantly increased. Similarly, the awareness of lung fibrosis as a risk factor increased from 36.4% to 100% (*p* < 0.001). No significant changes were observed for cigarette smoking (*p* = 0.999) or carcinogenic substances (*p* = 0.248).

**Table 2:** Knowledge regarding lung cancer.

	Before	After	*p*
	N = 154	N = 154	
Lung cancer is one of the most common cancers	1 (0.65%)	154 (100%)	<0.001
Lung cancer is one of the most common causes of death	151 (98.1%)	154 (100%)	0.248
Cigarette smoking is a risk factor for lung cancer	153 (99.4%)	153 (99.4%)	0.999
Shisha smoking is a risk factor for lung cancer	56 (36.4%)	153 (99.4%)	<0.001
E-cigarette smoking is not a risk factor for lung cancer	1 (0.65%)	153 (99.4%)	<0.001
Repeated and long-term exposure to passive smoking is a risk factor for lung cancer	129 (83.8%)	154 (100%)	<0.001
Exposure to harmful radiation is a risk factor for lung cancer	142 (92.2%)	154 (100%)	0.001
Exposure to asbestos is a risk factor for lung cancer	53 (34.4%)	154 (100%)	<0.001
Exposure to carcinogenic substances is a risk factor for lung cancer	151 (98.1%)	154 (100%)	0.248
Exposure to radon gas is a risk factor for lung cancer	56 (36.4%)	154 (100%)	<0.001
Lung fibrosis is a risk factor for lung cancer	56 (36.4%)	154 (100%)	<0.001
Family history of lung cancer is a risk factor for lung cancer	141 (91.6%)	154 (100%)	0.001
Air pollution is a risk factor for lung cancer	43 (27.9%)	154 (100%)	<0.001
Alcohol consumption is a risk factor for lung cancer	1 (0.65%)	153 (99.4%)	<0.001

Data were summarized using counts and percentages; The chi-squared test of independence was used for statistical testing.

The awareness of lung cancer screening and early detection was significantly improved post-campaign (Table 3). Correct responses regarding the role of early detection in improving prognosis increased from 90.9% to 100% (*p* < 0.001), and the recognition of its role in reducing mortality increased from 96.1% to 100% (*p* = 0.030). Misconceptions about screening were notably corrected, with responses to “Only smokers should proceed to do a screening test” shifting from 0.65% to 100% (*p* < 0.001) and those regarding the awareness that MRI is not used for lung cancer screening improving from 0.65% to 100% (*p* < 0.001).

Recognition that annual screening is required for current smokers aged 50–80 increased from 35.1% to 100% (*p* < 0.001), and awareness that former smokers within the past 15 years should also be screened rose from 35.1% to 100% (*p* < 0.001). The understanding of general screening eligibility improved, however, responses to “Everyone must screen for lung cancer” increased from 0.65% to 29.9% (*p* < 0.001), indicating possible overgeneralization. No significant differences were observed in awareness of low-dose CT as the correct screening method (*p* = 0.999) or in the recognition of the necessity of screening individuals with a 20-pack-year smoking history (*p* = 0.248).

A heatmap was used to visualize the percentage distribution of the responses to knowledge-based questions concerning lung cancer risk factors, and the distribution iscategorized as (a) before and (b) after the public health campaign. The color intensity within each cell represents to the percentage of respondents selecting a given answer choice (“No”, “I don’t know”, or “Yes”) regarding whether a specific factor is a risk for lung cancer. Darker shades of blue indicate higher agreement rates for a “Yes” response, whereas lighter shades and white indicate lower percentages. Each row represents a distinct risk factor question (e.g., “Alcohol consumption is a risk factor for lung cancer”). The data are summarized by showing the percentage of correct answers for each question before and after the campaign, allowing for a direct visual comparison of the knowledge improvement.

**Figure 1:**
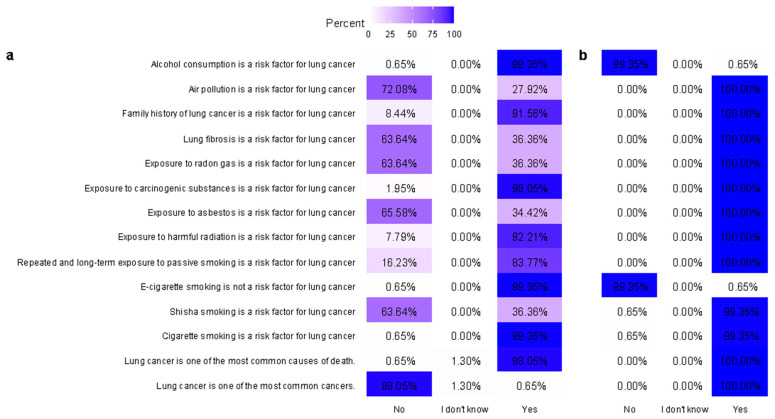
Responses to questionnaire knowledge items (**a**) before and (**b**) after the campaign in the matched cohort.

Before the campaign (Figure 1a), knowledge was variable, with misconceptions evident in several areas. Notably, only 72.08% recognized air pollution as a risk factor, and the awareness of lung fibrosis (63.64%), radon gas exposure (63.64%), and asbestos exposure (65.58%) as risk factors was relatively low. Additionally, only 16.23% correctly identified passive smoking as a risk factor, and a high percentage incorrectly believed that alcohol consumption is a risk factor (with 0.65% incorrectly disagreed).

After the campaign (Figure 1b), there was a marked improvement across all items, with most responses reaching 99.35–100% agreement for correct statements. Misconceptions were significantly corrected, particularly regarding e-cigarettes, air pollution, and passive smoking exposure.

The knowledge and awareness scores regarding lung cancer significantly improved after the campaign (*p* < 0.001). The awareness score increased from a median of 4.00 [4.00; 6.00] before the campaign to 9.00 [9.00; 10.00] after the campaign. Similarly, the median knowledge score rose from 7.00 [6.00; 9.00] to 14.00 [14.0; 14.0]. The median total score, representing overall knowledge and awareness, exhibited a substantial increase from 11.0 [10.0; 15.0] to 23.0 [23.0; 24.0]. The internal consistency was high across all scales, with values of 0.88 for awareness, 0.82 for knowledge, and 0.93 for the total score, indicating strong reliability. The Mann–Whitney U test confirmed the significant differences between the pre- and post-campaign scores (Table 4).

Linear regression analysis was used to examine the factors associated with knowledge scores before and after the campaign (Table 5). Before the campaign, being in the 30–39 age group was associated with a significantly lower knowledge score compared to the 20–29 age group (β = −9.11, 95% CI: −12.05 to −6.18, *p* < 0.001). Males had significantly lower scores than females (β = −7.11, 95% CI: −10.39 to −3.83, *p* < 0.001). Higher education (bachelor’s vs. secondary) was strongly associated with higher knowledge scores (β = 16.96, 95% CI: 12.09 to 21.83, *p* < 0.001), while smoking status (*p* = 0.128) and income level (*p* = 0.121) were not significant predictors. The model explained a substantial proportion of the variance (R^2^ = 0.890, adjusted R^2^ = 0.886).

After the campaign, the effect sizes of significant predictors were reduced. The 30–39 age group continued to have lower scores than the 20–29 age group (β = −2.79, 95% CI: −3.39 to −2.20, *p* < 0.001). Males still scored lower than females (β = −3.52, 95% CI: −4.27 to −2.77, *p* < 0.001), but the impact of education was no longer significant (*p* = 0.495). Smoking was now significantly associated with lower knowledge scores (β = −1.04, 95% CI: −1.41 to −0.66, *p* < 0.001), as was having an income above SAR 5000 (β = −3.04, 95% CI: −3.74 to −2.33, *p* < 0.001). The model explained a lower proportion of the variance post-campaign (R^2^ = 0.686, adjusted R^2^ = 0.675), suggesting that additional unmeasured factors influenced the knowledge scores after the intervention. It is important to note that certain categorical variables, such as gender (specifically the small number of female respondents before the campaign) and some education levels (e.g., respondents with only secondary education), had very limited subgroup sizes in the regression analysis. While statistical significance was observed for some of these predictors, the low number of observations in these specific categories could result in less stable and less generalizable coefficient estimates. Therefore, the interpretation of these particular effects should be approached with caution.

## 4. Discussion

Lung cancer remains one of the leading causes of cancer-related deaths worldwide, and this is largely due to late-stage diagnoses and the limited awareness of the risk factors and screening options [11]. This study’s findings demonstrate the effectiveness of a targeted awareness campaign in significantly improving knowledge of lung cancer risk factors, screening eligibility, and preventive measures. However, the persistence of some misconceptions, particularly with respect to universal screening recommendations, highlights the challenges of effectively translating awareness into actionable behavioral change. This discussion contextualizes the findings within the broader literature, compares them to similar awareness campaigns, and explores the implications for future public health initiatives. The significant post-campaign improvements in knowledge with respect to lung cancer risk factors align with prior research indicating that public health campaigns can effectively increase awareness and correct misconceptions [12]. In particular, this study exhibited drastic increases in awareness regarding alternative tobacco products, such as shisha and e-cigarettes, which were previously underestimated as risk factors. Similar findings were reported by Hinde and colleagues, who observed that many individuals incorrectly believe that non-cigarette forms of smoking pose little or no risk for lung cancer [13]. Furthermore, the increase in the awareness of environmental and occupational risk factors such as radon gas, asbestos, and air pollution is significant. Previous studies have indicated that public awareness of these risk factors is generally low [12]. A survey in the UK reported that less than 40% of participants could identify radon exposure as a risk factor for lung cancer [14]. Although the results of this study—where awareness increased from 36.4% to 100%—suggest that campaign messaging with respect to environmental risks was particularly effective, studies have also shown that increased awareness does not necessarily result in behavioral change. For example, increased awareness may not result in a reduction in exposure to secondhand smoke or improved workplace safety practices [15]. The observed increase in lung cancer screening awareness is consistent with previous studies examining the impact of educational campaigns. Nevertheless, future research should investigate whether participants who report improved knowledge actually modify their behaviors based on the information that they received. Despite the improvements in lung cancer awareness observed in this study, misconceptions remain prevalent, particularly in the Middle East and the Gulf region. Cultural beliefs, misinformation, and insufficient targeted public health education have contributed to persistent misunderstandings regarding lung cancer risk factors. For instance, many individuals in this region believe that waterpipe (shisha) smoking is significantly less harmful than cigarette smoking, despite evidence suggesting that it exposes users to higher levels of toxicants, including tar and carbon monoxide, which increase the lung cancer risk [16,17]. Additionally, studies have indicated that some populations in the Gulf region mistakenly associate lung cancer risks primarily with genetic predisposition rather than modifiable lifestyle and environmental factors [18]. This misconception may contribute to lower engagement in preventive measures such as smoking cessation or environmental exposure reduction. Another key issue is the misunderstanding of lung cancer screening recommendations, which was reflected in this study’s findings. Similarly to other research in the region, a substantial proportion of individuals mistakenly believed that everyone should be screened for lung cancer, rather than only high-risk groups such as long-term smokers aged 50–80 [19]. This misconception not only leads to unnecessary strain on healthcare systems but also raises concerns about false-positive results and associated patient anxiety. Research in Qatar and the UAE has also identified a general fear of cancer diagnoses, which discourages participation in screening programs [20]. Addressing these deep-rooted beliefs through culturally adapted awareness campaigns, community engagement, and physician-led education is crucial to improving early detection and reducing lung cancer mortality rates in the region. Globally, the “Be Clear on Cancer” campaign in the UK demonstrated that increasing knowledge of low-dose CT (LDCT) screening significantly improved uptake among high-risk individuals [21]. Similarly, a study in Wales found that a media-driven campaign successfully increased screening participation among individuals over 50 with a history of heavy smoking [22]. However, this study also identified a major issue of overgeneralization regarding screening eligibility. Post-campaign, nearly 30% of the participants incorrectly believed that everyone should be screened for lung cancer. This finding is concerning because unnecessary screening can result in false positives, anxiety, and the overuse of medical resources [14]. Campaigns should avoid overgeneralization and instead encourage individuals to consult healthcare providers about their specific risk factors. The effect of sociodemographic characteristics was evident in the current study. Given that smokers had lower post-campaign knowledge scores, additional efforts should focus on this demographic to address cognitive biases and misinformation. Gender-specific differences were also notable, with men exhibiting lower knowledge levels post-campaign. Thus, tailored messaging such as using social media campaigns featuring male influencers may increase engagement [22]. While our study offers important insights into a lung cancer awareness campaign’s effectiveness, its findings should be considered with respect to several limitations. First, because this was an interventional cross-sectional study, we could only capture immediate changes in knowledge after the campaign. We were unable to track whether these knowledge gains persisted over time or resulted in actual behavioral changes, such as quitting smoking or seeking screening advice. Second, despite our campaign’s success in boosting knowledge, a notable number of participants still misunderstood universal lung cancer screening guidelines. This suggests that our communication methods need refining, especially when targeting a population that does not meet the current screening criteria, highlighting the difficulty in simplifying complex medical information for broad campaigns. Third, our newly developed questionnaire, while assessed for internal consistency and reviewed by experts for content and face validity, did not undergo full psychometric validation. This means that the interpretation of our awareness and knowledge scores should be approached with caution. Finally, the small number of participants in certain demographic subgroups (such as specific education levels, women, and current smokers) within our regression analysis limits how broadly we can apply our results and the stability of our findings. Even when statistically significant, the results for these smaller groups should be interpreted carefully, as they might not truly represent the larger population. Future research would benefit from larger, more varied participant groups in order to better explore these demographic differences.

## 5. Conclusions

In conclusion, this study confirms that lung cancer awareness campaigns can significantly improve public knowledge about risk factors and screening. However, the emergence of new misconceptions, particularly regarding universal screening, highlights the importance of precise messaging. Future campaigns should emphasize shared decision-making, address gender and socioeconomic disparities, and use long-term educational approaches to ensure that awareness translates into action. These findings align with the existing literature while also highlighting areas for improvement in public health outreach strategies.

## Figures and Tables

**Table 3:** Awareness regarding lung cancer screening.

Awareness	Before	After	*p*
	N = 154	N = 154	
Early detection of lung cancer will contribute to a good prognosis	140 (90.9%)	154 (100%)	<0.001
Early detection of lung cancer can help with decreasing the mortality rate	148 (96.1%)	154 (100%)	0.030
Only smokers should proceed to do a screening test	1 (0.65%)	154 (100%)	<0.001
Lung cancer screening is done by MRI	1 (0.65%)	154 (100%)	<0.001
Lung cancer screening is done by low-dose CT	153 (99.4%)	154 (100%)	0.999
Everyone must screen for lung cancer	1 (0.65%)	46 (29.9%)	<0.001
Adults aged 50–80 who have a 20 pack-year or more smoking history should be screened for lung cancer	151 (98.1%)	154 (100%)	0.248
Adults aged 50–80 who are currently smoking should be screened annually for lung cancer	54 (35.1%)	154 (100%)	<0.001
Anyone who is smoking should screen annually for lung cancer	1 (0.65%)	153 (99.4%)	<0.001
Adults aged 50–80 who quit smoking within the past 15 years should be screened for lung cancer	54 (35.1%)	154 (100%)	<0.001

Data were summarized using counts and percentages; The chi-squared test of independence was used for statistical testing.

**Table 4:** Knowledge and awareness scores regarding lung cancer.

Scale	Knowledge Score Before Campaign	Knowledge Score After Campaign	*p*	
Awareness	4.00 [4.00; 6.00]	9.00 [9.00; 10.00]	<0.001	0.88
Knowledge	7.00 [6.00; 9.00]	14.00 [14.0; 14.0]	<0.001	0.82
Total	11.0 [10.0; 15.0]	23.0 [23.0; 24.0]	<0.001	0.93

Data were summarized using median/IQR; analysis was performed using the Mann–Whitney U test.

**Table 5:** Factors associated with the total score.

Predictor	Before Campaign			After Campaign		
	β	95% CI	*p*	β	95% CI	*p*
Age 20–29	Ref			Ref		
Age 30–39	−9.11	−12.05–−6.18		−2.79	−3.39–−2.20	
Gender [Male vs. Female]	−7.11	−10.39–−3.83		−3.52	−4.27–−2.77	
Education [Bachelor vs. Secondary]	16.96	12.09–21.83		−0.21	−0.80–0.39	0.495
Smoking [Yes vs. No]	1.16	−0.34–2.65	0.128	−1.04	−1.41–−0.66	
Income						
< SAR 5000	Ref					
> SAR 5000	−2.00	−4.54–0.54	0.121	−3.04	−3.74–−2.33	<0.001
R^2^/R^2^ adjusted	0.890/0.886			0.686/0.675		

## Data Availability

Data are contained within the article.

## Appendix A

**Table A1:** Descriptive statistics for all dataset.

	Before	After	*p*. Overall
	N = 713	N = 859	
Gender:			0.005
Female n (%)	421 (59.0%)	445 (51.8%)	
Male n (%)	292 (41.0%)	414 (48.2%)	
Age:			<0.001
18–19 n (%)	0 (0.00%)	30 (3.49%)	
20–29 n (%)	357 (50.1%)	473 (55.1%)	
30–39 n (%)	356 (49.9%)	277 (32.2%)	
40–49 n (%)	0 (0.00%)	79 (9.20%)	
Education:			<0.001
Secondary n (%)	390 (54.7%)	100 (11.6%)	
Bachelor’s n (%)	323 (45.3%)	589 (68.6%)	
Higher Education n (%)	0 (0.00%)	170 (19.8%)	
Smoking:			<0.001
No n (%)	293 (41.1%)	578 (67.3%)	
Yes n (%)	420 (58.9%)	281 (32.7%)	
Monthly income:			<0.001
	620 (87.0%)	156 (18.2%)	
< SAR 5000, n (%)	92 (12.9%)	377 (43.9%)	
SAR 5000–10,000, n (%)	1 (0.14%)	232 (27.0%)	
SAR 11,000–20,000, n (%)	0 (0.00%)	94 (10.9%)	

Significant differences were observed between the pre-campaign (N = 713) and post-campaign (N = 859) groups. The gender distributions differed, with a higher proportion of females in the pre-campaign group (%) compared to the post-campaign group (59.0% vs. 51.8%, *p* = 0.005). The age distribution also varied, with more participants aged 30–39 in the pre-campaign group (%) compared to the post-campaign group (49.9% vs. 32.2%, *p* < 0.001). The educational levels differed, with a higher percentage of participants having a secondary education in the pre-campaign group (54.7%) compared to the post-campaign group (11.6%), while the post-campaign group had a larger proportion with higher education (19.8%) (*p* < 0.001). The smoking status differed, with a higher proportion of smokers in the pre-campaign group (58.9%) compared to the post-campaign group (58.9% vs. 32.7%, *p* < 0.001). The monthly income distributions varied significantly, with 87.0% of the pre-campaign group earning less than SAR 5000, compared to 18.2% in the post-campaign group (*p* < 0.001).

**Table A2:** Knowledge regarding lung cancer.

Question	Before (N = 713)	After (N = 859)	*p*-Value
Lung cancer is one of the most common cancers.	1 (0.14%)	859 (100%)	<0.001
Lung cancer is one of the most common causes of death.	447 (62.7%)	859 (100%)	<0.001
Cigarette smoking is a risk factor for lung cancer.	681 (95.5%)	855 (99.5%)	<0.001
Shisha smoking is a risk factor for lung cancer.	610 (85.6%)	855 (99.5%)	<0.001
E-cigarette smoking is not a risk factor for lung cancer.	12 (1.68%)	855 (99.5%)	<0.001
Repeated and long-term exposure to passive smoking is a risk factor for lung cancer.	512 (71.8%)	859 (100%)	<0.001
Exposure to harmful radiation is a risk factor for lung cancer.	535 (75.0%)	859 (100%)	<0.001
Exposure to asbestos is a risk factor for lung cancer.	91 (12.8%)	859 (100%)	<0.001
Exposure to carcinogenic substances is a risk factor for lung cancer.	447 (62.7%)	859 (100%)	<0.001
Exposure to radon gas is a risk factor for lung cancer.	305 (42.8%)	859 (100%)	<0.001
Lung fibrosis is a risk factor for lung cancer.	283 (39.7%)	859 (100%)	<0.001
Family history of lung cancer is a risk factor for lung cancer.	386 (54.1%)	859 (100%)	<0.001
Air pollution is a risk factor for lung cancer.	59 (8.27%)	859 (100%)	<0.001
Alcohol consumption is a risk factor for lung cancer.	682 (95.7%)	118 (13.7%)	<0.001

Correct response rates significantly improved across all survey questions after the awareness campaign (*p* < 0.001 for most items). The recognition of lung cancer as a common disease increased from 0.14% to 100% (*p* < 0.001), and the awareness of its status as a leading cause of death increased from 62.7% to 100% (*p* < 0.001). The understanding of cigarette smoking as a risk factor increased from 95.5% to 99.5% (*p* < 0.001), while the awareness of shisha smoking as a risk factor improved from 85.6% to 99.5% (*p* < 0.001). The awareness of the dangers of e-cigarette smoking experienced the most pronounced shift, rising from 1.68% to 99.5% (*p* < 0.001). Knowledge of environmental and occupational risk factors also improved, with correct responses for passive smoking exposure increasing from 71.8% to 100% (*p* < 0.001), asbestos exposure from 12.8% to 100% (*p* < 0.001), and radon gas exposure from 42.8% to 100% (*p* < 0.001). Similarly, the recognition of lung fibrosis as a risk factor increased from 39.7% to 100% (*p* < 0.001). The awareness of air pollution as a risk factor increased from 8.27% to 100% (*p* < 0.001). However, the misconception that alcohol consumption is a risk factor for lung cancer decreased from 95.7% to 13.7% (*p* < 0.001).

**Table A3:** Awareness regarding lung cancer screening.

Question	Before (N = 713)	After (N = 859)	*p*-Value
Early detection of lung cancer will contribute to a good prognosis.	414 (58.1%)	859 (100%)	<0.001
Early detection of lung cancer can help with decreasing the mortality rate.	604 (84.7%)	859 (100%)	<0.001
Only smokers should proceed to do a screening test.	43 (6.03%)	853 (99.3%)	<0.001
Lung cancer screening is done by MRI.	41 (5.75%)	829 (96.5%)	<0.001
Lung cancer screening is done by low-dose CT.	712 (99.9%)	859 (100%)	0.454
Everyone must screen for lung cancer.	712 (99.9%)	509 (59.3%)	<0.001
Adults aged 50–80 who have a 20 pack-year or more smoking history should be screened for lung cancer.	447 (62.7%)	859 (100%)	<0.001
Adults aged 50–80 who are currently smoking should be screened annually for lung cancer.	92 (12.9%)	859 (100%)	<0.001
Anyone who is smoking should screen annually for lung cancer.	672 (94.2%)	124 (14.4%)	<0.001
Adults aged 50–80 who quit smoking within the past 15 years should be screened for lung cancer.	92 (12.9%)	859 (100%)	<0.001

Awareness of lung cancer screening and early detection improved substantially after the campaign. The proportion of participants recognizing that early detection improves prognosis increased from 58.1% to 100% (*p* < 0.001), while the awareness of its role in reducing mortality rose from 84.7% to 100% (*p* < 0.001). Misconceptions about screening eligibility were significantly corrected, with a decrease from 6.03% to 99.3% with respect to the recognition that “Only smokers should proceed to do a screening test” (*p* < 0.001). Similarly, the awareness that MRI is not a valid screening method for lung cancer improved from 5.75% to 96.5% (*p* < 0.001). The understanding of appropriate screening eligibility criteria also showed improvement. The awareness that adults aged 50–80 with a 20-pack-year smoking history should undergo screening increased from 62.7% to 100% (*p* < 0.001). Recognition that current smokers in this age group should undergo annual screening improved from 12.9% to 100% (*p* < 0.001), and awareness that former smokers who quit within the past 15 years should also be screened increased from 12.9% to 100% (*p* < 0.001). However, misconceptions remained regarding universal screening, as the percentage of participants believing that “Everyone must screen for lung cancer” remained high at 59.3% after the campaign (*p* < 0.001). Additionally, while the awareness of the need for screening among smokers improved, the proportion of participants incorrectly believing that all smokers should be screened annually decreased but remained high (94.2% to 14.4%, *p* < 0.001).
